# Complete Genome Sequence of *Rhodococcus* sp. B7740, a Carotenoid-Producing Bacterium Isolated from the Arctic Sea

**DOI:** 10.1128/genomeA.00333-15

**Published:** 2015-04-30

**Authors:** Di Zhang, Li Li, Sidong Zhu, Ning Zhang, Jifang Yang, Xiangdong Ma, Jigang Chen

**Affiliations:** a College of Fisheries and Life Sciences, Shanghai Ocean University, Shanghai, China; b College of Biological and Environmental Sciences, Zhejiang Wanli University, Ningbo, China; c Hubei Collaborative Innovation Center for Green Transformation of Bio-Resources, Hubei University, Wuhan, China

## Abstract

*Rhodococcus* sp. B7740 was isolated from Arctic seawater and selected for its capacity to synthesize carotenoids. Here, we report the complete genome sequence of *Rhodococcus* sp. B7740 to provide the genetic basis for a better understanding of its carotenoid-accumulating capabilities, and we describe the major features of the genome.

## GENOME ANNOUNCEMENT

Carotenoids are noted for their antioxidant and anticancer abilities, as well as for boosting the immune system and for other effects. Carotenoids have therefore attracted extensive attention as a natural food additive (1). Microbial fermentation is currently the healthiest and easiest way of producing carotenoids, though production of wild strains of microorganisms still needs to be improved. Increasing research has recently focused on the modification of wild strains of microorganisms by genetic engineering in order to increase their production of carotenoids (2), including metabolic engineering of *Rhodococcus* (3). *Rhodococcus* sp. B7740 is a psychrotolerant, carotenoid-producing bacterium isolated from Arctic seawater (4). We report the complete genome sequence of *Rhodococcus* sp. B7740 as the basis for determining the pathways involved in carotenoid production.

*Rhodococcus* sp. B7740 was cultured in the optimized medium (beef extract 7 g, yeast extract 5 g, glucose 5 g, lactose 3 g, seawater 1 liter). Genomic DNA was prepared using a DNA extraction kit (BioTech) following the manufacturer’s instructions. Complete genome sequencing was performed by Nextomics Biosciences Co., Ltd. (Wuhan, China) using single-molecule real-time sequencing (SMRT) technology. Genomic DNA was interrupted randomly and repaired to construct a DNA library, which was then qualified using an Agilent 2100 Bioanalyzer high-sensitivity kit. The DNA fragments were then sequenced using a PacBio RS II SMRT DNA sequencing system (5, 6). Finally, the reads were assembled using Hierarchical Genome Assembly Process 2.2.0 (6). Two SMRT cells were sequenced, and 1.04 GB of data were obtained. Reads from dozens up to 20,000 bp were preassembled using BLASR (7) alignment and Celera Assembler (8). Annotation was performed using the combined results from RAST (9) and GLIMMER version 3.0 (10). tRNAs and rRNAs were identified using the tRNAscan-SE (11), RNAmmer (12), and Rfam (13) databases. The genome sequence was then searched against the NCBI NR, Swiss-Prot, COG, tRRMBL, InterProScan, and KEGG protein databases to annotate the gene descriptions.

The complete genome comprised 5,341,557 bases, including 5,149 predicted coding sequences, with a G+C content of 64.93%. Forty-seven tRNAs, four 5S rRNAs, four large subunit rRNAs, and four small subunit rRNAs were detected in the genome. The genome encoded a total of 468 subsystems, some of which showed carbohydrate subsystem features. Analysis of the *Rhodococcus* sp. B7740 genome resulted in the identification of some key carotenoid-biosynthesis genes, including geranylgeranyl pyrophosphate synthetase, phytoene synthase, and phytoene dehydrogenase. A more detailed analysis of this genome and its comparison with the genomes of other carotenoid-producing bacteria may provide further insight into the mechanisms of carotenoid synthesis.

### Nucleotide sequence accession number. 

The complete genome sequence of *Rhodococcus* sp. B7740 has been deposited in GenBank under the accession no. CP010797.
